# A multi-subject, multi-modal human neuroimaging dataset

**DOI:** 10.1038/sdata.2015.1

**Published:** 2015-01-20

**Authors:** Daniel G Wakeman, Richard N Henson

**Affiliations:** 1 Athinoula A. Martinos Center for Biomedical Imaging, Charlestown, Massachusetts 02129, USA; 2 MRC Cognition & Brain Sciences Unit, Cambridge CB2 7EF, England

**Keywords:** Electroencephalography - EEG, Brain imaging, Functional magnetic resonance imaging, Cognitive neuroscience

## Abstract

We describe data acquired with multiple functional and structural neuroimaging modalities on the same nineteen healthy volunteers. The functional data include Electroencephalography (EEG), Magnetoencephalography (MEG) and functional Magnetic Resonance Imaging (fMRI) data, recorded while the volunteers performed multiple runs of hundreds of trials of a simple perceptual task on pictures of familiar, unfamiliar and scrambled faces during two visits to the laboratory. The structural data include T1-weighted MPRAGE, Multi-Echo FLASH and Diffusion-weighted MR sequences. Though only from a small sample of volunteers, these data can be used to develop methods for integrating multiple modalities from multiple runs on multiple participants, with the aim of increasing the spatial and temporal resolution above that of any one modality alone. They can also be used to integrate measures of functional and structural connectivity, and as a benchmark dataset to compare results across the many neuroimaging analysis packages. The data are freely available from https://openfmri.org/.

## Background & Summary

There have been many advances in non-invasive neuroimaging of humans, in attempts to better understand the working of the human brain. The most dominant methods are magnetic resonance imaging (MRI: both structural, sMRI, and functional, fMRI) and electro- and magneto- encephalography (EEG and MEG respectively). fMRI provides good spatial resolution (on the order of 1 mm^1^), but poor temporal resolution (on the order of 1 s), given that it is an indirect hemodynamic (metabolic) consequence of brain activity^2^. EEG and MEG provide a more direct measure of neural activity with exquisite temporal resolution (on the order of 1 ms), but poor spatial information (on the order of 1 cm), because of the difficulty of localising activity within the brain from data recorded outside the head (the so-called ‘M/EEG inverse-problem’). Given the complementary strengths of these modalities, much recent effort has been devoted towards methods for multi-modal integration.

Though EEG and MEG are sensitive to the same generators (synchronous post-synaptic currents in aligned pyramidal cells), they provide somewhat complementary information. For example, MEG cannot detect the radial component of current sources, while EEG is more sensitive to the precise geometry and conductivity of the head (which is difficult to model). Indeed, certain combinations of sources result in signal cancellation either exclusively in EEG, exclusively in MEG, or in both^3^. Furthermore, different sensor-types within each modality have different sensitivities, such as the magnetometers and planar gradiometers used for MEG here, which are more sensitive to deep versus superficial sources respectively. Therefore, simultaneously inverting data from multiple EEG and MEG sensor-types can be more accurate than inverting any one sensor-type alone^4–9^.

Given the indeterminacy of the M/EEG inverse problem, further assumptions (constraints) are needed. For example, fMRI data can be used to constrain the localisation of M/EEG data^10–12^. Given that the generators of the fMRI and M/EEG signals are unlikely to be identical though^13^, most approaches use fMRI data as ‘soft’ constraints, such as priors in probabilistic Bayesian frameworks^11,14–19^.

Both M/EEG and fMRI are improved through the incorporation of sMRI data. For example, the M/EEG inverse problem benefits from accurate models of the cortex, skull and scalp, in order to approximate effects of the different conductivities of these tissue-types on the propagation of electromagnetic fields. These surfaces are often extracted from a T1-weighted image. This can be helped by further MR contrasts like Proton Density (PD), which can be calculated for example from Multi-Echo Fast Low Angle SHot (MEFLASH) sequences. Better MR contrast between skull and CSF in particular allows more accurate estimation of the inner skull surface. There is also the potential to measure the electrical conductivity throughout the head, for which diffusion-weighted MR images of brains may be helpful^20^. Finally, fMRI data can also benefit from the incorporation of sMRI information. For example, fMRI of the cortex can often yield more accurate results when grey matter is properly segmented from an sMRI image and analyzed on that surface^21^.

There is also much recent interest in functional connectivity between brain regions, as measured for example by fMRI and M/EEG. Indeed, common frameworks exist for estimating connectivity across these modalities (e.g., Dynamic Causal Modelling^22^). Furthermore, estimates of functional connectivity can be informed by information about structural connectivity (e.g., from Diffusion-Tensor Imaging, DTI).

Different individuals also have different neuroanatomies and possibly different functional-anatomical mappings. The value of simulations based on a single head model is therefore limited. Moreover, pooling data across individuals can help constrain MEG/EEG localisation^23^. Here we provide data from 19 healthy young individuals. Though a relatively small number of individuals, in that a greater number would be needed to fully address individual differences (e.g., as a function of age), this number is typical of many neuroimaging studies. There is also considerable measurement noise within functional data from a single individual, which is normally addressed by averaging across multiple trials. Here we provide hundreds of trials, for each of three conditions, offering comparisons with both a high (for faces versus scrambled faces) and low (for familiar versus unfamiliar faces) signal-to-noise-ratio (SNR). These trials are grouped into multiple runs, also allowing estimation of test-retest reliabilities.

Finally, there are many software packages for analysing neuroimaging data (see Usage Notes), yet multimodal benchmark datasets are rare. We therefore hope that the provision of multiple modalities from multiple runs on multiple individuals will prove valuable in this respect.

## Methods

### Participants

All research was conducted in accordance with the Declaration of Helsinki (1964). Participants were members of the MRC Cognition & Brain Sciences Unit participant panel who responded to an invitation to participate. 19 people completed both MEG and MRI visits, of which 8 were female, and 11 were male, with an age range 23–37 years. All were Caucasian except for one Asian participant, who had spent many years in the UK.

The study was approved by Cambridge University Psychological Ethics Committee. Written informed consent was obtained from each participant prior to and following each phase of the experiment. Participants also gave separate written consent for their anonymized data to be freely available on the internet. The first 16 participants undertook the MEG experiment during their first visit, while the remaining 3 undertook the MRI first. All had a gap of approximately three months between their first and second visits.

### Stimuli

The face stimuli comprised two sets of 300 greyscale photographs, half from famous people and half from nonfamous people (unknown to participants), compiled from previous sources^24^ (the image files are also supplied with data, though note that, due to an error, the identity of famous face images f076.bmp and f117.bmp is identical). Half of the faces were male, half female. The famous faces were selected in order to be recognized by the majority of British adults. Nonfamous faces were approximately matched to famous faces in terms of their sex and age (by visual judgment). All photos were matched and cropped to show only the face. The photos covered a wide range of hairstyles (though long hair was cropped), expressions (though mainly happy or neutral), and orientations (though all were taken from between a full-frontal to 3/4-view perspective).

There were two photographs of each face (one for each set), taken from slightly different viewpoints, lighting conditions, etc. One set of photographs was used for the M/EEG recording session, and the other was used for the fMRI recording session. While use of faces of the same people across the two visits may induce neural effects of that repetition, such effects are unlikely to affect differences between the conditions^25^. Each scrambled face was generated from either the famous face or the non-famous face of the same stimulus number. These were scrambled by taking the 2D-Fourier transform of the faces, permuting the phase information, and then inverse-transforming back into the image space. To match the overall approximate shape and size of the original faces, the scrambled images were finally cropped to a mask created by a combination of one famous and one nonfamous face. Although this masking meant that the power density spectrum of the scrambled faces no longer exactly matched that of the original faces, matching the visual angle of the two types of stimuli was deemed more important, e.g., to minimize differences in eye-movements.

### Experimental design

The basic design of the experiment is based on^25^. Stimuli were projected onto a screen approximately 1.3 m in front of the participant, subtending horizontal and vertical visual angles of approximately 3.66° and 5.38° respectively. The photographs were presented against a black background, with a white fixation cross in the center.

The start of a trial was indicated by the appearance of a fixation cross for a random duration between 400 and 600 ms, after which the critical stimulus (face or scrambled face) was superimposed for a random duration between 800 and 1,000 ms. The random jitter before stimulus onset was to reduce aliasing of ongoing neural oscillations (at least above approximately 5 Hz) and to avoid any pre-stimulus phase resetting. The offset jitter was to average out visual offset effects (again, at least above 5 Hz). The interstimulus interval comprised a central white circle for 1,700 ms. Participants were told to fixate centrally throughout the experiment, with the change from central circle to central cross helping to prepare the participant for each stimulus. They were also instructed to try to not blink during the cross-hair or stimulus (but to blink freely during the circle).

Each image was presented twice, with the second presentation occurring either immediately after (Immediate Repeats), or after 5–15 intervening stimuli (Delayed Repeats), with 50% of each type of repeat. The reason for manipulating this repetition lag was because it has been shown to modulate repetition-related effects^26^. For the technical validation here however, this repetition manipulation is ignored (i.e., initial and repeated presentations are not distinguished), leaving 3 trial-types (conditions): Familiar (Famous) Faces, Unfamiliar (Nonfamous) Faces and Scrambled Faces.

To ensure attention to each stimulus, participants were asked to press one of two keys with either their left or right index finger (assignment counter-balanced across participants). Their key-press was based on how symmetric they regarded each image: pressing one or the other key depending whether they thought the image was ‘more’ or ‘less symmetric’ than average. The range of symmetries (hence idea of average symmetry) was made apparent from a practice session of 23 separate photos (not used in the main experiment). Participants continued this practice until they were comfortable with the task. The reason for using this task was because it can be performed equally well on face and non-face stimuli^25^. Because this symmetry judgment is somewhat subjective however, there were no behavioral data of interest (other than mean reaction times, which were typically 955 ms with a standard deviation 283 ms).

### M/EEG acquisition

Both the MEG and EEG were measured in a light magnetically shielded room using an Elekta Neuromag Vectorview 306 system (Helsinki, FI). Five head-position indicator (HPI) coils were attached to the EEG cap and stimulated with sinusoidal currents at 293, 307, 314, 321, and 328 Hz. A 70 channel Easycap EEG cap (based on EC80 system here: http://www.easycap.de/easycap/e/products/products.htm#15) was used to record the EEG data simultaneously, with electrode layout conforming to the extended 10–10% system. A 3D digitizer (Fastrak Polhemus Inc., Colchester, VA, USA) was used to record the locations of the EEG electrodes, the HPI coils and approximately 50–100 ‘head points’ along the scalp, relative to three anatomical fiducials (the nasion and left and right pre-auricular points). The EEG location was digitized by inserting the digitizer pen into the paste in the middle of the donut-shaped electrodes until the pen touched the scalp.

Following the practice, stimuli were presented in six, 7.5 min runs. Data were acquired at an 1100 Hz sampling rate with a lowpass filter at 350 Hz and no highpass filter.

The EEG reference electrode was placed on the nose, and the common ground electrode was placed at the left collar bone. Two sets of bipolar electrodes were used to measure vertical (left eye) and horizontal electro-oculograms (VEOG and HEOG), and another set was used to measure the electro-cardiogram (ECG): left lower rib and right collarbone (due to a problem with the acquisition software, these three channels are not properly labelled in the fif files: EEG061=HEOG; EEG062=VEOG; EEG063=ECG). Twenty seconds of data without continuous HPI were collected at the start of each run prior to beginning the stimulus program. A fixed 34 ms delay exists between the appearance of a trigger in the MEG file (on channel STI101) and the appearance of the stimulus on the screen.

A small number of empty-room recordings acquired around the same dates as the participants’ data are also available with the dataset (these can be used to estimate characteristics of the environmental noise in the MEG sensors).

After the M/EEG acquisition, participants saw each of the 300 faces again, but now used three buttons to indicate whether (1) they had not seen the face before the experiment, (2) the face looked familiar, but they could not remember from where, or (3) they knew the face, i.e. could remember definite fact about them, such as their job, a movie they were in, their name etc. On average across participants, 73% of famous faces were given a rating of 2–3, and 86% of nonfamous faces were given a rating of 1. These debriefing data could be used to further refine familiarity of each participant with each face, and are available on request, but were not used in the current validation.

### M/EEG pre-processing

The continuous MEG data from each run were processed with MaxFilter 2.2 (Elekta Neuromag), which entailed: (i) fitting a sphere to the digitized head points, excluding those on the nose, and using the center of this sphere, together with location of sensors, to define a spherical harmonic basis set for Signal Space Separation (SSS) in order to remove environmental noise^27^, (ii) automatic detection of bad channels throughout the run (range across participants and runs was from 0 to 14, median=2), (iii) notch-filtering of the 50 Hz line-noise and its harmonics, and of the HPI coil signals (from 293–321 Hz), (iv) compensating for movement every 10 ms within each run, (v) aligning the data across runs to match the head position at the start of the fourth run (the range across participants of movement between runs was 0.2 to 7.9 mm, median=1.9 mm). Note that the temporal extension of SSS was not employed, nor was any transformation to a default space. Both the raw data and the maxfiltered data are provided, though the analyses illustrated here use the maxfiltered data.

### MRI acquisition

The MRI data were collected from a Siemens 3T TIM TRIO (Siemens, Erlangen, Germany). A standard 1 mm isotropic T1-weighted ‘structural’ image was acquired using an MPRAGE sequence (TR 2,250 ms, TE 2.98 ms, TI 900 ms, 190 Hz/pixel; flip angle 9°). The face of the participant in the T1 image was subsequently manually removed to help maintain anonymity. The functional data were acquired using an EPI sequence of 33, 3 mm-thick axial slices (TR 2000 ms, TE 30 ms, flip angle 78°). Slices were acquired in an interleaved fashion, with odd then even numbered slices (where slice 1 was the most inferior slice) and a 25% distance spacing (increased where necessary to cover whole of cortex), resulting in a range of voxel sizes of 3×3×3.75 mm to 3×3×4.05 mm across participants. 210 volumes were acquired in each of 9 runs (note that the 3 initial TRs were discarded to allow saturation of T1 effects).

Two bandwidth-matched Multi-Echo FLASH sequences (1 mm isotropic; 651 Hz/pixel; TR 20 ms) were also acquired on each participant, at both 5° and 30° flip angles for each of 7 echo-times (TE 1.85 ms; 4.15 ms; 6.45 ms; 8.75 ms; 11.05 ms; 13.35 ms; 15.65 ms), as was a Field Map EPI sequence (TE 5.19 and 7.65 ms) for potential mapping of distortions. On 13 of the participants, a diffusion-weighted EPI sequence (64 directions with 2 mm isotropic voxels) was also acquired on a separate visit.

The trial-timing during the functional runs was slightly different from the MEG experiment, in that 6 blocks of 20 s of fixation were interspersed after every 50 s of stimuli, in order to be able to estimate the BOLD response versus ‘baseline’.

### MRI pre-processing

The DICOM images were converted to NIFTI format.

## Data Records

The data are freely available on OpenfMRI (Data Citation 1). This resource contains 20 downloadable archive (tar) files: one for each of the 19 subject’s data (approximately 7.9GB compressed) and one called ‘metadata’ (approximately 710MB compressed) with further information about the data, including a ‘README’ text file that explains the data structure in more detail. For the purposes of this section, we describe the Scientific Datasets approved repository (www.openfmri.org) layout. The dataset follows their layout (for complete details please see https://openfmri.org/content/data-organization). Due to the number of special data types included in this dataset, we have expanded on this structure.

Starting in the metadata tar file, we have an additional 3 directories: stimuli, models, and empty_room. In the stimuli directory, we have two different subdirectories: meg and mri. Within each of these are the bmp files presented to the participants in each of the experiments. The models directory contains another subdirectory: model001 with an OpenfMRI condition_key.txt file and a task_contrasts.txt file. The empty_room directory contains three different empty-room MEG measurements from dates near the dates that the MEG data were collected. These (like all of the MEG data in this study) are provided in two Neuromag FIF files, one of which has been maxfiltered (as described above). These files are named yymmdd_raw.fif.gz and yymmdd_raw_st.fif.gz (where yy refers to the last two digits of the year the data was collected and the mm refers to the month and the dd refers to the day).

Each subject directory has six additional subdirectories: behav, model, BOLD, diffusion, anatomy, and MEG. The behav directory contains a file called behavioral.txt with the behavioral responses of participants after performing the MEG portion of the experiment. The model directory contains the OpenfMRI structure for specifying the onsets of the Faces and Scrambled images: model/model001/onsets/task001_run00*/cond00*.txt. The BOLD directory contains one directory for each fMRI run titled task001_run00*. In each of these directories is a text file which has complete information about all of the stimuli presented and the participants responses titled task001_run00*.txt. The BOLD data are in a file called bold.nii.gz. Additionally, all of the OpenfMRI testing files for the BOLD data are in each of these directories. To add to this data we also include three gzipped NIFTI files that can be used to construct a fieldmap to reduce BOLD EPI distortion, with the magnitude volumes labelled fm_m_X.nii.gz with the X indicating the echo (0 or 1) and the phase volumes labelled fm_p.nii.gz. The BOLD directory also contains a T1-weighted EPI image to assist with data registration (titled t1_epi.nii.gz). The diffusion directory (only present for 13 of the 19 participants) contains a dwi.nii.gz file as well as two text files titled dwi.bval (containing 65 B values) and dwi.bvec (containing 65 gradient directions). The anatomy directory contains the MPRAGE titled highres001.nii.gz (as well as some OpenfMRI post-processing files). Inside the anatomy directory is a FLASH directory with 14 MEFLASH volumes (from two different sequences), labelled meflash_XX_XX.nii.gz (the first XX are numbers corresponding to the flip angle of the volume in degrees and the second XX correspond to the echo number, starting at 00). The MEG directory contains two Neuromag FIF files for each of the 6 runs, one before MaxFiltering, labelled run_0X_raw.fif and one after MaxFiltering, labelled run_0X_sss.fif (for Signal Space Separation), which contain the MEG, EEG, EOG and ECG data. Additionally, a text file labelled subject_**_X.txt provides the details of the exact stimulus presented at each trigger. One further file titled subject_**-trans.fif provides a mapping between the MRI data and the digitization data calculated by the researchers using the full face information (which has been removed in the MRI here for de-identification).

## Technical Validation

### M/EEG sensor analysis

To illustrate the type of results one can obtain from these data, and validate the basic data quality, we report a number of basic analyses on a subset of 16 participants (the subset used for the training set for the 2014 international Biomag conference; https://www.kaggle.com/c/decoding-the-human-brain/leaderboard). To analyse the MEG and EEG data, we used SPM8 (http://www.fil.ion.ucl.ac.uk/spm) with Matlab7 (MathWorks, Natick, MA, USA), but emphasize that many other software packages could be used (see Usage Notes). The continuous data in each run was first epoched from −500 to +1,200 ms around the onset of each stimulus. Across participants, between 880 and 889 epochs were extracted (approximately 295 per condition). These epochs were then lowpass filtered to 32 Hz, removing the initial and final 400 ms to accommodate filter artifacts (resulting in epochs from −100 to +800 ms), followed by manual detection of bad EEG channels (range across participants was 0 to 4, median=0) and re-referencing the EEG data to the average across all non-bad electrodes, concatenating the data across runs, rejecting trials in which the VEOG or HEOG exceeded 100 μV (range across participants was 0 to 153 rejected trials, median=46), and then averaging the remaining trials for each of the three conditions (range across participants and conditions of remaining valid trials was 225 to 296, median=280).

**Figure 1:**
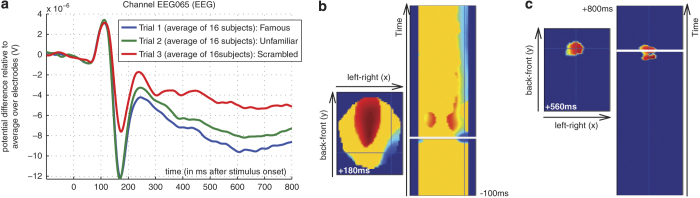
Example ERP data. (**a**) shows grand-average over participants of ERPs to familiar, unfamiliar and scrambled faces at a right posterior electrode. Note negative deflection around 170 ms (N170) greater for faces than scrambled faces, while from around 250 ms, there is a divergence for familiar relative to unfamiliar faces. (**b**) shows three orthogonal sections through a 3D statistical map over a space-time volume created after each participant’s ERP data were topographically projected onto the scalp and interpolated onto a 2D grid for each time sample, and then the statistical contrast of faces versus scrambled faces evaluated across participants for each voxel in these volumes (see text). Only voxels that survived correction using random field theory are shown (nonsignificant voxels in yellow; warm colors mean a significant positive difference and cold colors a significant negative difference). (**c**) shows the same thing for the contrast of familiar versus unfamiliar faces.

Figure 1a shows the grand average Event Related Potential (ERP) from a right parieto-occipital electrode. A negative deflection peaking around 170 ms (‘N170’ component) is larger for faces than scrambled faces, but does not differ for familiar and unfamiliar faces. Around 250 ms, a slower potential shift distinguishes familiar and unfamiliar faces until the end of the epoch. A similar ‘M170’ component was seen for the MEG data (magnetometers and gradiometers; data not shown).

For statistical analysis across participants, a mass univariate General Linear Model (GLM) was used, corresponding to a one-way (1×3), repeated-measures ANOVA. Statistical Parametric Maps (SPMs) of the F-statistic (given that polarity was not of interest) were created for two, orthogonal planned comparisons: (1) Faces versus Scrambled faces, averaging across Familiar and Unfamiliar faces, and (2) Familiar versus Unfamiliar faces.

Figure 1b shows statistical tests of the ERP difference between Faces and Scrambled faces across all time points and scalp locations, where the data from each EEG sensor are projected from 3D space to a flat surface and interpolated to a 48×48 pixel grid for each sample (~0.9 ms). These planes are then tiled for every sample from −100 to +800 ms to create a 3D volume, over which Random Field Theory^28^ can be applied to correct for multiple comparisons across space and time, after Gaussian smoothing by 5 mm in-plane and 5 ms across time. The suprathreshold voxels characterize spatiotemporal clusters of when and where (topographically) significant differences are found. The Face versus Scrambled face differences are significant from around 160 ms, and continue to the end of the epoch, with a topographic distribution over fronto-central electrodes (more positive for Faces) and lateral parieto-occipital electrodes (more negative for Faces). The Familiar versus Unfamiliar face difference is relatively weaker in terms of signal to noise, so the threshold in Fig. 1c is reduced to *P*<0.001 uncorrected (with minimum cluster size of 1,000), after which there is only a single cluster over midfrontal electrodes from 520–620 ms (more Positive for Familiar faces). Similar SPMs are found for the magnetometer data and/or (RMS of) the planar gradiometer data (not shown), though with topographic distributions depending on the sensor-type. These results demonstrate the basic signal quality of the present data.

**Figure 2:**
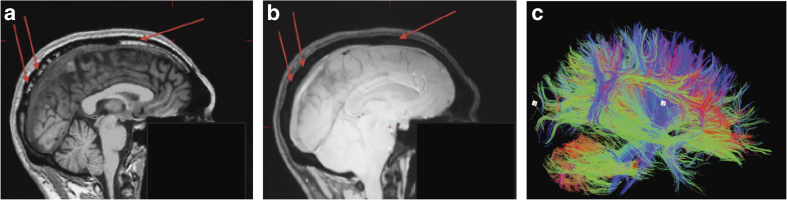
Example MRI data. (**a**) shows a sagittal section through T1-weighted image of one participant from the MPRAGE sequence (face removed), while (**b**) shows the same section through same participant using a contrast optimised for skull-brain contrast from the ME-FLASH sequence. The red arrows indicate tissue that makes it difficult to extract the inner skull/CSF boundary in the MPRAGE image but not the ME-FLASH image. (**c**) shows an example of the major white-matter tracts that can extracted from the Diffusion-Weighted data from the same participant.

The data from EEG electrodes, MEG magnetometers and MEG planar gradiometers can be combined via simultaneously inverting the forward-model for each sensor-type. The forward models are created by first creating a head-model that captures the scalp, inner-skull, outer-skull and cortical surfaces. These can be extracted direct from sMRI images, such as the T1-weighted image from the MPRAGE sequence (Fig. 2a). Note however that the inner-skull boundary is not as clearly defined as with a contrast optimised for the relevant tissue-types that can be derived from the ME-FLASH sequence (Fig. 2b). For the present analyses however, we simplify the process of head-model construction by using scalp/skull/cortical surfaces that have already been created for a template brain in SPM8, and warping these ‘canonical’ surfaces to match the brains of each of the individual participants^29^. The warps are determined via spatially normalising each participant’s T1-weighted image to the T1-weighted image of the template brain (see below). This template-warping approach avoids the difficulties associated with automatically extracting an accurate cortical surface from an sMRI image, given the convoluted nature of that surface (though see ref. 30 for effective alternatives). However, more accurate cortical and scalp/skull surfaces could be obtained directly from individual ME-FLASH images in future.

The MEG and EEG data were coregistered with the T1 MRI image by using (1) the fiducial positions marked on the T1-weighted sMRI and digitized during the M/EEG experiment and (2) the scalp surface and the additional head-points digitized during the M/EEG experiment. The resulting coregistered ‘canonical’ meshes and sensor positions were then used to create a forward-model (leadfield matrix) using a single-shell deformed-sphere approximation^31^ for the MEG data and a three-shell Boundary Element Model (BEM) for the EEG data. In theory, more accurate forward-models could be obtained by using information from the Diffusion-weighted images (Fig. 2c), e.g., to quantify anisotropies of volume conduction. The cortical sources of the M/EEG data across the whole epoch for each condition were estimated using Multiple Sparse Priors^16^. The power of the source estimate for each condition between 0–32 Hz and from 0–800 ms was then calculated for each vertex, and interpolated to a 3D MNI space of 2 mm×2 mm×2 mm voxels. Finally, the 3D images were smoothed with an isotropic 3D Gaussian with a FWHM of 8 mm (to allow for residual inter-participant differences), and entered into the same ANOVA model as above for the sensor-space statistics.

**Figure 3:**
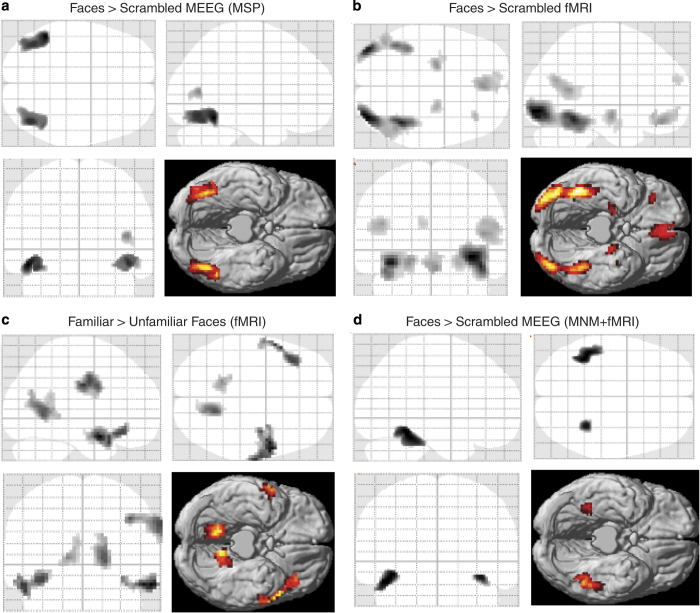
Example of Multimodal integration. (**a**) shows Maximal Intensity Projections (MIPs), together with a rendering on the ventral cortical surface of a standard brain in Montreal Neurological Institute (MNI) space, of voxels where faces produced significantly greater power between 0–32 Hz and 0–800 ms than scrambled faces across participants, after simultaneously localising both the EEG and MEG data, and thresholding to correct for multiple comparisons using random field theory. Note the bilateral ventral and right lateral occipitotemporal clusters (see text). (**b**) shows the corresponding results for the same statistical test on the fMRI data. (**c**) shows where familiar faces produced greater BOLD response than unfamiliar faces at *P*<0.001 uncorrected. (**d**) shows where faces produced greater source-localised power than scrambled faces after using the major fMRI clusters from (**b**) to inform the localisation of the EEG and MEG data (see text).

Figure 3a shows the results in a glass-brain in MNI space for the T-contrast for greater energy for Faces than Scrambled faces, thresholded at *P*<0.001 uncorrected. The left and right clusters in posterior fusiform/ventral occipital cortex survive correction for multiple comparisons in their spatial extent, and represent regions that would be expected to show face-related activity based on previous studies (and are also consistent with the fMRI results reported below). There is an additional but weaker cluster in right posterior, superior temporal cortex, which is also consistent with previous studies (e.g., intracranial EEG) and likely to be captured in particular by the EEG data^9^. This is an example of full data fusion (symmetric integration) of MEG and EEG data^19^.

### fMRI analysis

The volumes with Blood Oxygenation Level Dependent (BOLD) contrast were realigned within and across runs to compensate for movement and then the data corrected for different slice acquisition times by synchronizing with the middle slice. The mean BOLD image across volumes was coregistered with the T1 image, which was then segmented and spatially normalized to the MNI template space, and the resulting warps applied to all the BOLD volumes, interpolating to 3 mm isotropic voxels. The data were then smoothed by a 3D 8 mm isotropic Gaussian kernel.

Statistical analysis within each participant used a linear convolution GLM applied to the timeseries within each voxel concatenated across the 9 runs. The BOLD response was modelled by convolving a delta function at the onset of each stimulus with a canonical hemodynamic response function (HRF) with 16 simulated time-points per volume. The resulting time-courses were down-sampled at the middle time-point per volume to form the GLM regressors for each of the 3 conditions of interest, with separate sets of regressors for each run. A further 6 regressors per run were added that captured the rigid-body movement parameters from the above spatial realignment step (in order to capture residual movement artifacts). Voxel-wise parameter estimates for these regressors were obtained by Restricted Maximum-Likelihood (ReML) estimation, using a temporal high-pass filter (cut-off 128 s) to remove low-frequency drifts, and modelling temporal autocorrelation across scans with an AR(1) process. Contrasts of these parameter estimates were created in order to average each condition across the 9 runs, and these averaged images were entered into the same GLM as for the M/EEG data above.

Figure 3b shows the results of the group fMRI analysis, specifically voxels showing greater BOLD response to Faces than Scrambled faces, thresholded at *P*<0.001 uncorrected. Here there are distinct fusiform (FFA) and occipital (OFA) peaks in both hemispheres, plus a cluster in right posterior, superior temporal cortex (all of which survive correction for multiple comparisons), similar to those reconstructed with MEEG in Fig. 3a. There are also further fMRI clusters in anterior medial temporal lobes and orbitofrontal cortex. Figure 3c shows instead the (orthogonal) contrast of greater BOLD response for Familiar than Unfamiliar faces (at same threshold). This includes bilateral temporal pole clusters, extending to inferior prefrontal cortex on left, and bilateral medial parietal cortex as expected from previous fMRI and neuropsychological studies^25^.

Finally, Fig. 3d shows another example of multi-modal integration, here combining the fMRI data with the M/EEG data, whereby the 4 largest fMRI clusters for the comparison of Faces and Scrambled Faces in Fig. 3b were used as separate spatial priors for the source reconstruction of the M/EEG data^12^, together with a standard minimum norm prior. This also illustrates further multi-participant integration, in that the weightings of the spatial priors were optimized across participants by aligning their lead-field matrices^23^. The result, while still not having the spatial resolution of fMRI, is a single bilateral cluster that is more anterior to that without fMRI priors (in Fig. 3a) and closer to the FFA in the fMRI data in Fig. 3b. The activity of these clusters can then be estimated at the millisecond timescale, which would not be possible with fMRI alone.

## Usage Notes

Some of the most common software packages for analysing these data are freely available, and include SPM (http://www.fil.ion.ucl.ac.uk/spm/), FieldTrip (http://fieldtrip.fcdonders.nl/), FSL (http://fsl.fmrib.ox.ac.uk/fsl/fslwiki/), FreeSurfer (http://surfer.nmr.mgh.harvard.edu/), MNE (http://martinos.org/mne/), Brainstorm (http://neuroimage.usc.edu/brainstorm/), EEGLAB (http://sccn.ucsd.edu/eeglab/), NIPY (http://www.nipy.org) and AFNI (http://afni.nimh.nih.gov/afni/). There are also several others (see http://en.wikipedia.org/wiki/List_of_neuroimaging_software for a more complete list). The Matlab scripts used for the Technical Validation in this paper can be provided by the authors.

We request only that researchers acknowledge Daniel Wakeman and Richard Henson in any publication arising from these data, and cite this paper and Data Citation 1 for the source of the data. These data are being integrated into the SPM12 manual as a multimodal demonstration, and into the MNE-python tutorials.

## Additional information

**Competing financial interests:** The manufacturer of the Elekta Neuromag system used to acquire the MEG and EEG data, Elekta Ltd, kindly contributed to the funding of the PhD studentship awarded to Daniel Wakeman, to develop new methods for MEG source reconstruction, and during which this test dataset was acquired. However, the manufacturer had no input on the choice of dataset, nor has any conflicting interest in the results or their publication. This dataset was awarded the 2010 Biomag conference Data Competition award.

**How to cite this article:** Wakeman, D. G. & Henson, R. N. A multi-subject, multi-modal human neuroimaging dataset. *Sci. Data* 2:150001 doi: 10.1038/sdata.2015.1 (2015).

## Supplementary Material



## References

[d1] OpenfMRI Wakeman D. G. Henson R. N. 2014 ds000117

